# Supplementary material for: Do Patients with Prostate Cancer Benefit from Exercise Interventions? A Systematic Review and Meta-Analysis
Source: Int J Environ Res Public Health. 2022 Jan 15;19(2):972. doi: 10.3390/ijerph19020972 (PMC8776086; doi:10.3390/ijerph19020972)
Supplement: Supplementary file 1 [file ijerph-19-00972-s001.zip › supplementary/File S1.pdf]

ESM 1 Database search strategies

1. Prostatic Neoplasms [mh]
2. Prostat*
3. (cancer* OR tumor* OR tumour* OR neoplas* OR malignan* OR adenocarcinoma* OR metasta*)
4. 1 OR (2 AND 3)
5. Physical activity[mh]
6. Exercise[mh]
7. Sports[mh]
8. Exercise Therapy[mh]
9. (exercise OR aerobic* OR resistance* OR strength* OR stretch* OR training* OR fitness* OR walk* OR endurance* OR lifestyle* OR sport* OR isometric* OR eccentric* OR plyometric* OR physical condition*)
10. (physical* AND (fit* OR activ*))
11. 5 OR 6 OR 7 OR 8 OR 9 OR 10
12. 4 AND 11
13. randomized controlled trial [pt]
14. controlled clinical trial [pt]
15. randomized [tiab]
16. placebo [tiab]
17. clinical trials as topic [mesh: noexp]
18. randomly [tiab]
19. trial [ti]
20. 13 OR 14 OR 15 OR 16 OR 17 OR 18 OR 19
21. 12 AND 20
22. Animals[mh] NOT humans [mh]
23. 21 NOT 22
