# Supplementary material for: Do Patients with Prostate Cancer Benefit from Exercise Interventions? A Systematic Review and Meta-Analysis
Source: Int J Environ Res Public Health. 2022 Jan 15;19(2):972. doi: 10.3390/ijerph19020972 (PMC8776086; doi:10.3390/ijerph19020972)
Supplement: Supplementary file 1 [file ijerph-19-00972-s001.zip › supplementary/File S3.pdf]

Online resource 3. Risk of bias with reason

Random sequence allocation

Twenty-eight trials were at low risk of selection bias as they adequately reported their randomised sequence with a random component [19-30,32-34,36,38-48,50].

Four trials had insufficient information on the randomisation sequence and were judged to have an unclear risk [31,35,37,49].

Allocation concealment

Twenty-two trials adequately concealed allocation to the intervention and control group, so neither participants nor investigators could identify the allocation. These trials were judged to have a low risk of selection bias [19-25,27-33,35,38-40,42-46,48,50].

Eight trials did not have sufficient information on methods of allocation concealment to permit an assessment, which is why they were considered to have an unclear risk [26,31,34,36,37,41,47,49]

Blinding of outcome assessors

Twenty-eight trials reported objective outcomes [19-24,26-37,39,40,43,44,46,47,49,50]. Of these, eight reported blinding techniques for objective outcome assessments and were judged to have a low risk of detection bias. Bjerre et al. [19] had staff to perform dual energy x-ray absorption (DXA) scans blinded to group allocation, while Bourke et al. [20] had all objective outcome assessors blinded. Focht et al. [27] reported that assessments of the primary and secondary outcomes were obtained by study staff blinded to the treatment arm, while Jones et al. [35] reported blinding of baseline and post-intervention assessments. Nilsen et al. [39] had staff conducting DXA scans blinded to allocation and a blinded statistician. Winters-Stone et al. [49] and Winters-Stone et al. [50] reported blinding of outcome assessors at baseline and follow-ups. Ndjavero et al. [38] had a blinded outcome assessor and data analyst, while Taaffe et al. [45] had blinded investigators and testing personnel.

Hojan et al. [32] and Hojan et al. [33] reported blinded statisticians and data managers but did not report information data collectors, which is why they were considered to have unclear risk. Six trials reported no blinding procedure of objective outcome assessors and were judged to have high risk. Bourke et al. [21] did not use blinding of outcome assessors in any objective outcome, while Galvao et al. [29] reported no blinding on

cardiovascular fitness and muscle strength. Eriksen et al. [26] reported no blinding of outcome assessors, and O'Neill et al. [40] reported no blinding of the outcome assessor. Uth et al. [46] and Hvid et al. [34] had neither assessor of physical function nor DXA scan blinded to group allocation. All other trials contained insufficient information to permit judgement, which why they were categorised as unclear risk [22-24,28,30,31,36,37,43,44,47].

Incomplete outcome data

Twenty trials reported analysing data according to intention-to-treat (ITT) principles. [19,22-24,27-31,35,36,38-40,42-45,48-50]. Of these, six trials had low dropout rates or less than 20% missing data and were judged to have a low risk of attrition bias [19,35,38,42,43,49,50]. Focht et al. [27] reported a dropout rate at 22% but used the last observation carried forward to account for missing data. Wall et al. [48] had high dropout in the control group (30%), but missing data were imputed using a linear mixed model and therefore judged to have low risk. The following ITT studies were judged to have high risk: Galvao et al. [29] reported 28% dropout in the intervention group, which was not balanced with the control group (16%); Cormie et al. [23] reported only 27% completeness in sexual function (EORTC QLQ-PR25) and had an unbalanced dropout rate; Taaffe et al. [44] had an imbalanced dropout rate between groups; Nilsen et al. [39] had 29% missing data in lower body muscle strength in the intervention group; and Taffe et al. [45] reported a high and imbalanced dropout rate. Two studies had high risk in objective outcomes: Cormie et al. [22] reported 40% missing data in the 400-meter walk test and muscle strength, while Livingston et al. [36] had a high dropout rate for objective outcomes (30%) presented in a secondary analysis by Gaskin et al. [60] as some participants did not allow use of objective data.

Twelve trials did not report data analysed according to [20,21,24-26,30,32,33,37,41,46,47], four of which had a high risk of attrition bias: Bourke et al. [20] had 33% dropout at the 12-month follow-up (6 months after the end of the intervention); Dawson et al. [24] reported ITT; however, as participants were excluded from analysis due to low adherence, the analysis was per protocol; and Monga et al. [37] and Park et al. [41] had a high dropout rate (30% and 23% respectively). Four studies had unclear attrition bias: Eriksen et al. [26] conducted per-protocol analysis and had moderate dropout (20%); Galvao et al. [30] analysed only complete cases due to bone pain and had an overall low dropout rate (18%), though they only analysed only 78% of the complete cases; Hojan et al. [32] had no ITT information but a low dropout rate (4%); and Hojan et al. [33] reported ITT but conducted an as-treated analysis, though a low dropout rate was observed (14%). The remaining four studies had a low risk of attrition bias: Bourke et al. [21] had no information on ITT (referred to CONSORT guidelines) but had low dropout rates (8%); Dierperink et al. [25] analysed only fully completed questionnaires

but had low dropout rates (4%); Windsor et al. [47] had a low dropout rate (2%); and Uth et al. [46] also had a low dropout-rate (18% in control).

Selective reporting

Twelve trials had a low risk of reporting bias. They were registered prospectively or had an available study protocol, just as there was consensus between prespecified outcomes and those published [19,22,23,28,29,33,34,39,40,43,48,49].

Fourteen trials were judged to have a high risk of reporting bias as they failed to report adverse events [25,26,31,32,36,37,38,42,45,47] or published outcomes different from those prescribed in the protocol [21,24,26,27,36,41,44,46]. Reporting adverse events should be standard in interventional clinical trials.

Two trials were considered to have an unclear risk, as no protocol or study registration had taken place [20,35]. Galvao et al. [30] only reported fatigue in the body of their study with the p-value but the results were not presented in a table, which is why this study was judged to have an unclear risk. Winters-Stone et al. [50] prespecified the outcome material quality, but this was not reported in the article, which means the study was considered to have a high risk.

Other biases

Three trials reported baseline differences and were judged to have a high risk of selection bias because the differences may affect outcome scores. Livingston et al. [36] reported an imbalance in cancer stage and cancer treatment, while O'Neill et al. [40] reported an imbalance in marital status and educational level. Bourke et al. [21] reported an imbalance in comorbidities such as cardiovascular disease and joint and bone deficits.

Three trials reported low adherence (<75%) to the intervention protocol or were excluded due to low adherence and were given a high risk of bias [19,24,48]. Park et al. [41] did not report adherence, and resistance exercise was only performed the last four weeks of the intervention, which the study was considered at high risk of bias.

Six trials had only partially described adherence, causing them to be judged as an unclear risk [22,23,25,27,28,30,38,45]. Two trials failed to report information on the imputation procedure of missing data, and therefore also judged to have unclear risk [42,43]. Another eleven trials were judged to have unclear risk of other bias due to following causes: We assume that Bourke et al. [20] had an interim analysis published in a feasibility study in 2011 [20]. Eriksen et al. [26] and Focht et al. [27] had no power calculation; however, these were pilot studies. Hvid et al. [34] also did not have a power calculation. Hebert et al. [31] reported a lower level

of physical activity in the intervention group at a six-month follow-up compared to baseline. Jones et al. [35] had a low follow-up at 12 months (70%) and had only 72% adherence to the home-based exercise protocol. Windsor et al. [47] do not clearly state whether the intervention lasted 4 or 8 weeks, which is why their data were not included in the analysis. Monga et al. [37] had insufficient information on adherence to exercise protocol. Uth et al. [46] had a baseline difference in body weight and height but without clinical importance. Winters-Stone et al. [49] failed to recruit a target trial sample.

Six trials were judged to have a low risk of other biases [29,32,33,39,44,50].
