# Supplementary material for: Do Patients with Prostate Cancer Benefit from Exercise Interventions? A Systematic Review and Meta-Analysis
Source: Int J Environ Res Public Health. 2022 Jan 15;19(2):972. doi: 10.3390/ijerph19020972 (PMC8776086; doi:10.3390/ijerph19020972)
Supplement: Supplementary file 1 [file ijerph-19-00972-s001.zip › supplementary/File S4.pdf]

**Online resource 4** Additional analyses to primary outcomes

## Cancer-specific Quality of Life

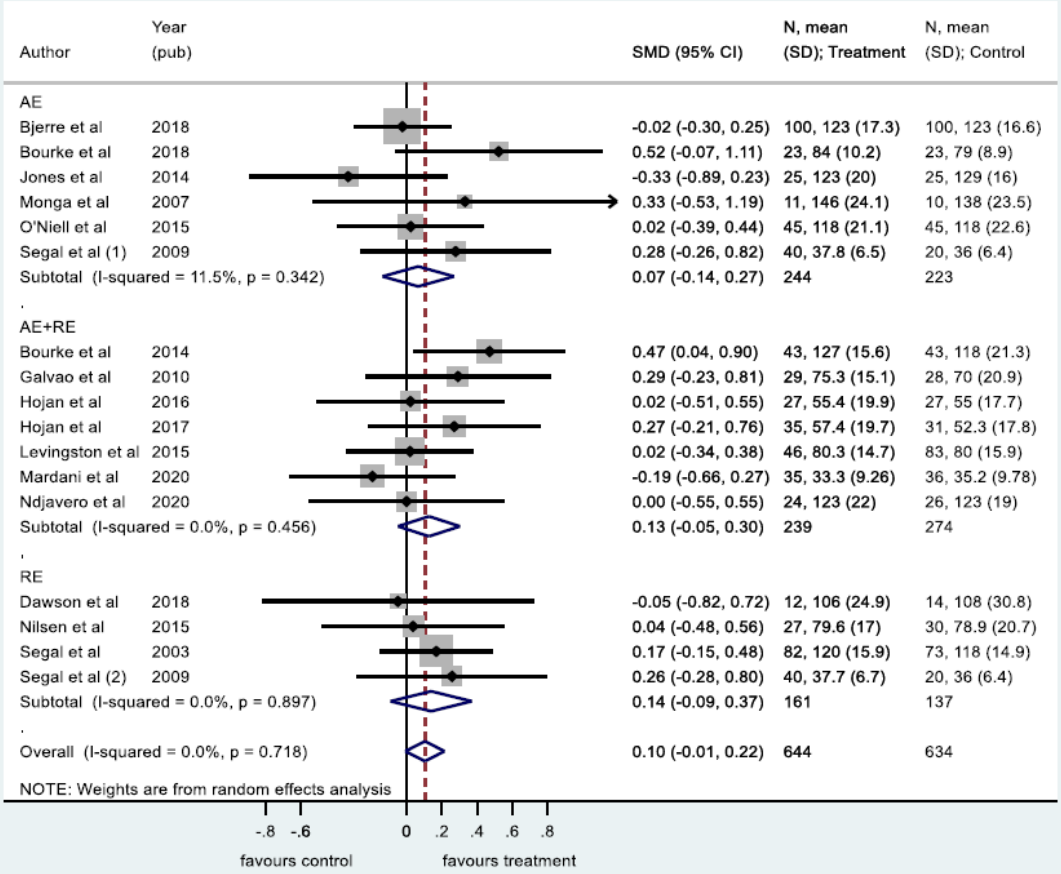


**Figure S1.** Pooled standard mean difference (SMD) on Cancer-specific quality of life comparing exercise interventions with usual care or control in men with prostate cancer stratified after exercise modality. A random effects model of DerSimonian & Laird, with estimate of heterogeneity from the Mantel-Haenszel model was used. AE: aerobic exercise, RE: resistance exercise.

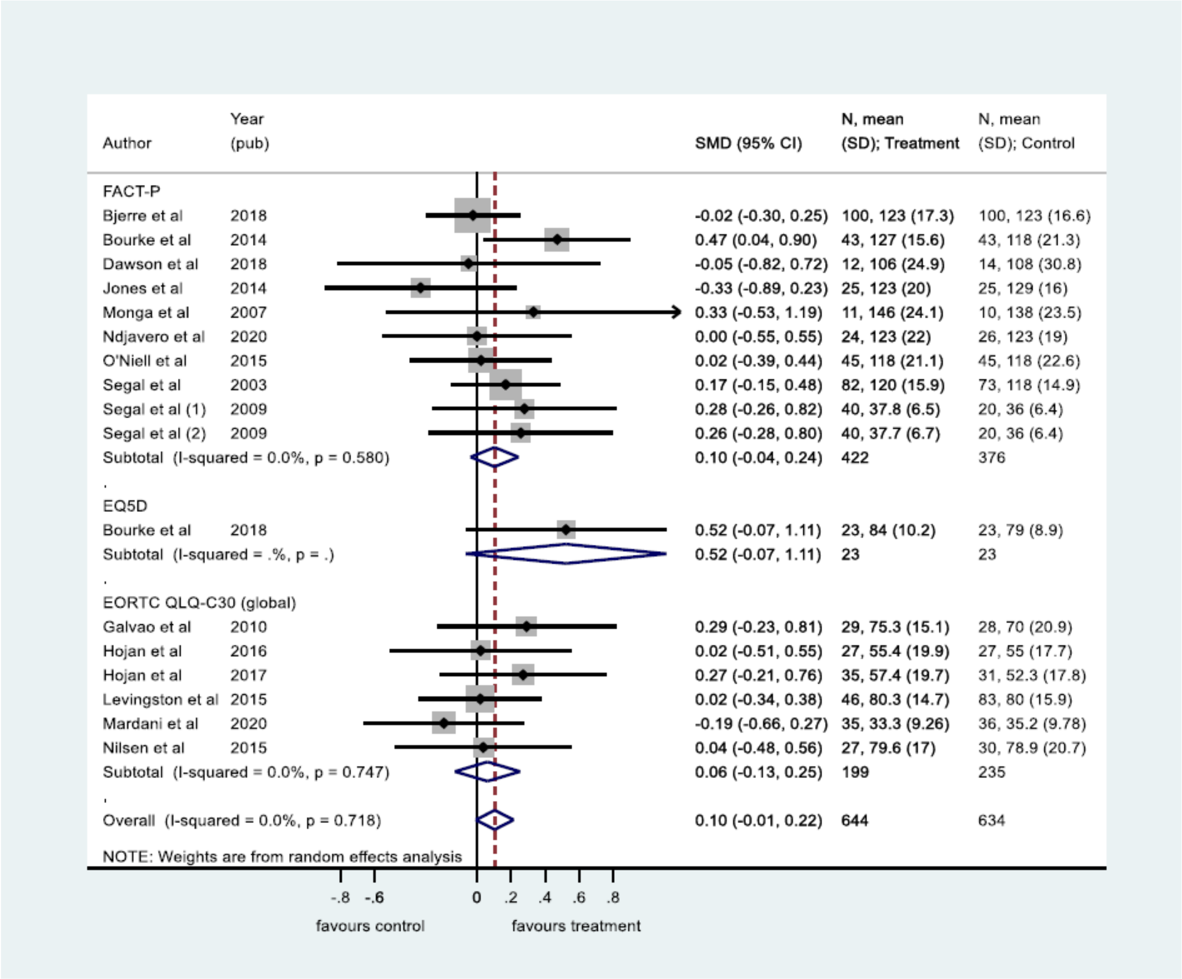


**Figure S2.** Pooled standard mean difference (SMD) on Cancer-specific quality of life comparing exercise interventions with usual care or control in men with prostate cancer stratified after assessment instrument. A random effects model of DerSimonian & Laird, with estimate of heterogeneity from the Mantel-Haenszel model was used.

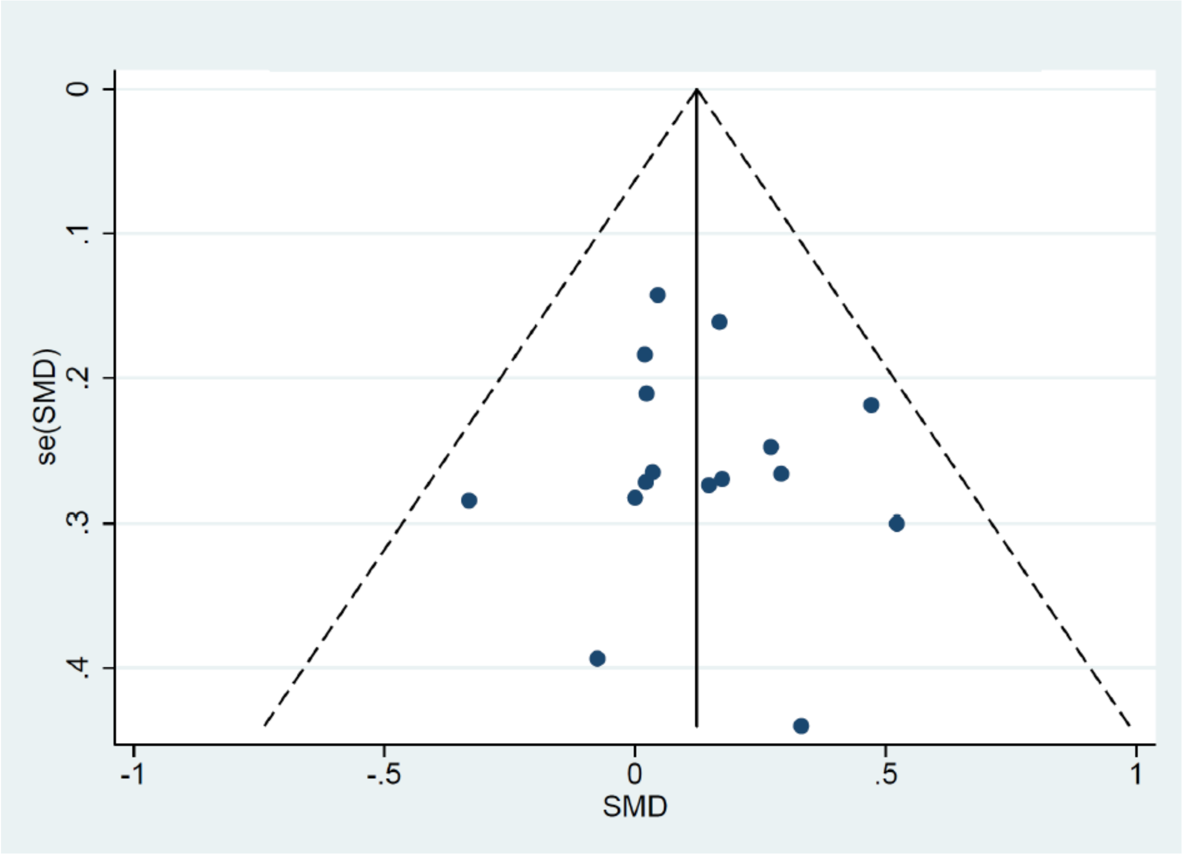


**Figure S3.** Funnel plot with pseudo 95% confidence limit onCancer-specific quality of life in men with prostate cancer. Egger's test showed no small-study effects (P=0.729).

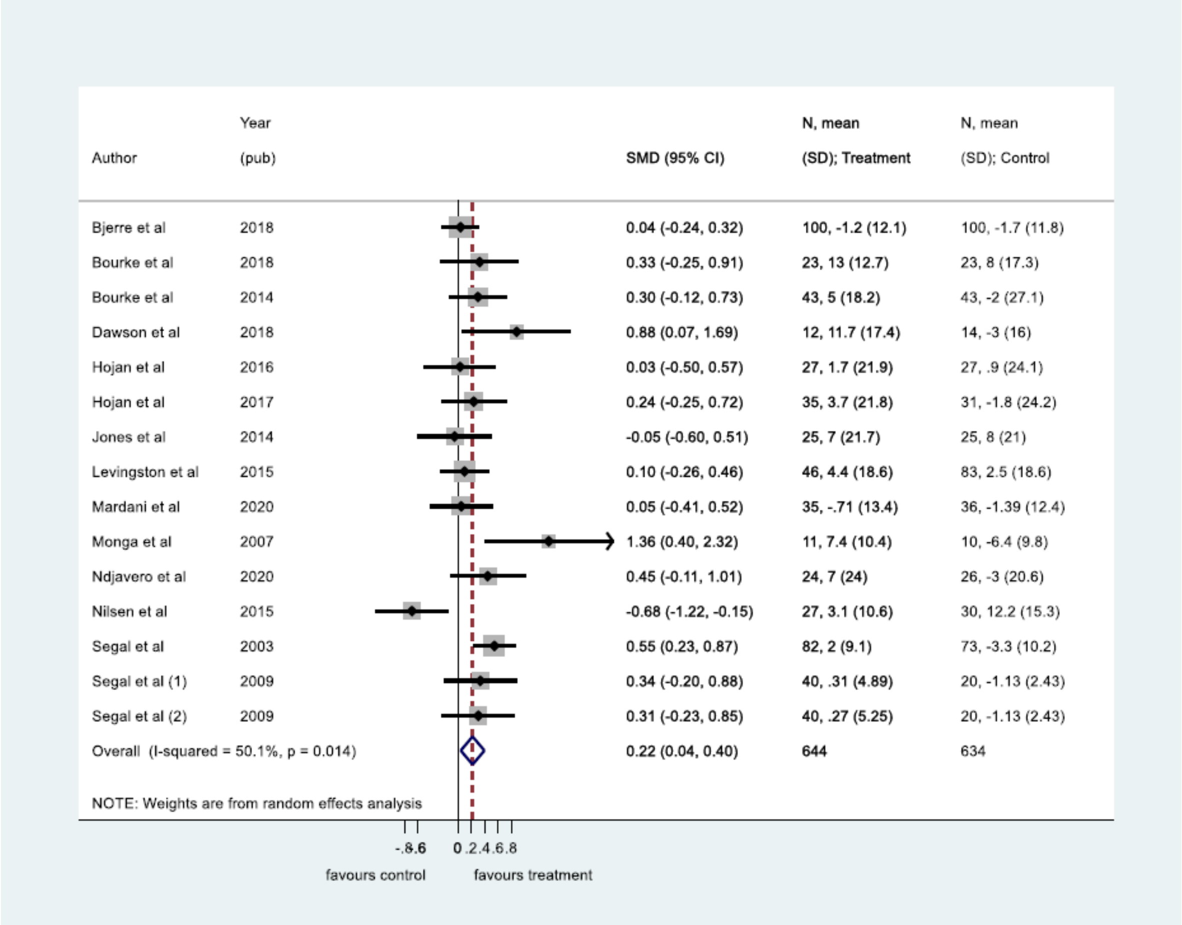

**Figure S4.** Pooled standard mean difference (SMD) for change score values on Cancer-specific quality of life comparing exercise interventions with usual care or control in men with prostate cancer. A random effects model of DerSimonian & Laird, with estimate of heterogeneity from the Mantel-Haenszel model was used.

## Cardiovascular fitness

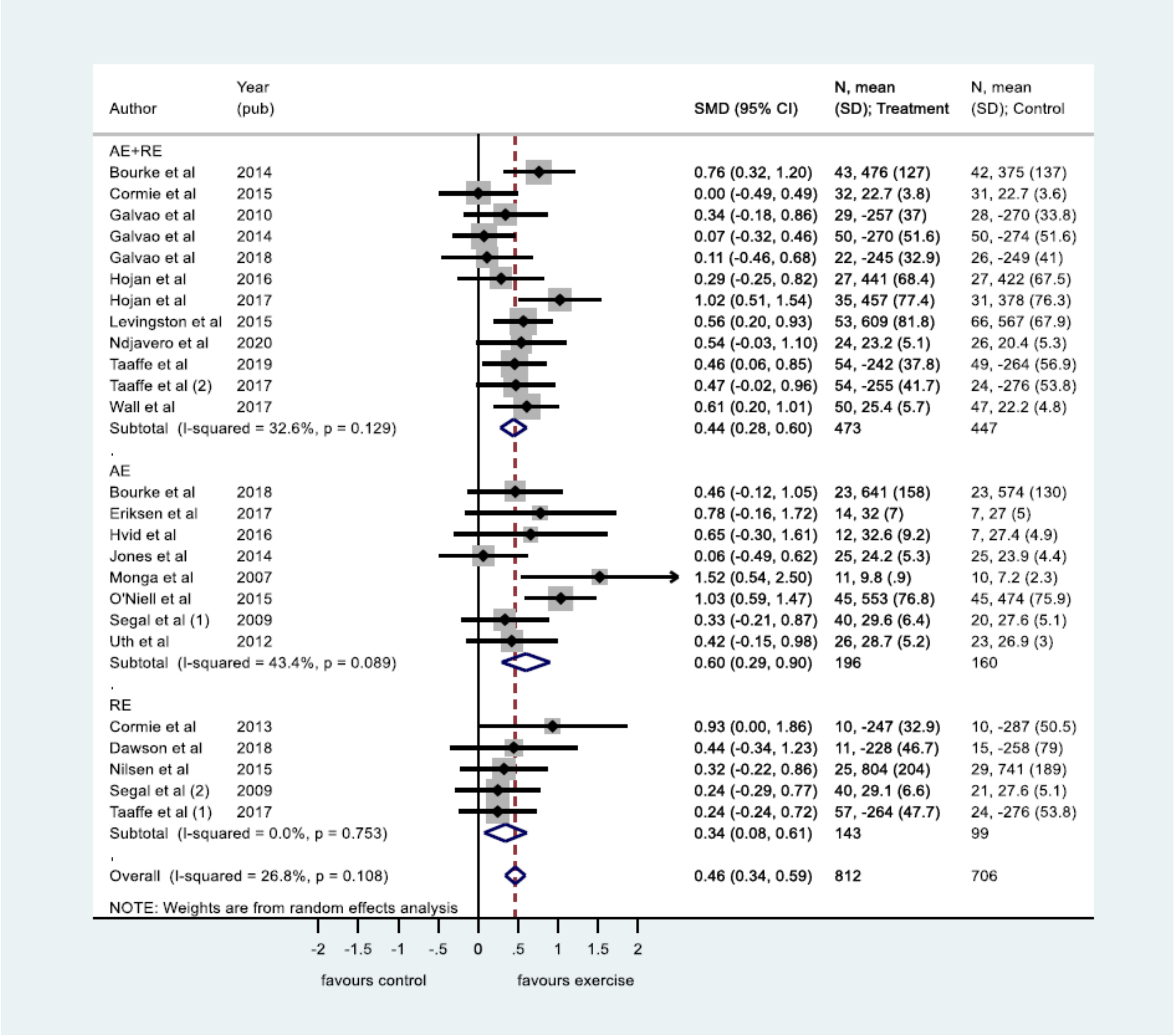


**Figure S5.** Pooled standard mean difference (SMD) on Cardiovascular fitness comparing exercise interventions with usual care or control in men with prostate cancer stratified after exercise modality. A random effects model of DerSimonian & Laird, with estimate of heterogeneity from the Mantel-Haenszel model was used.

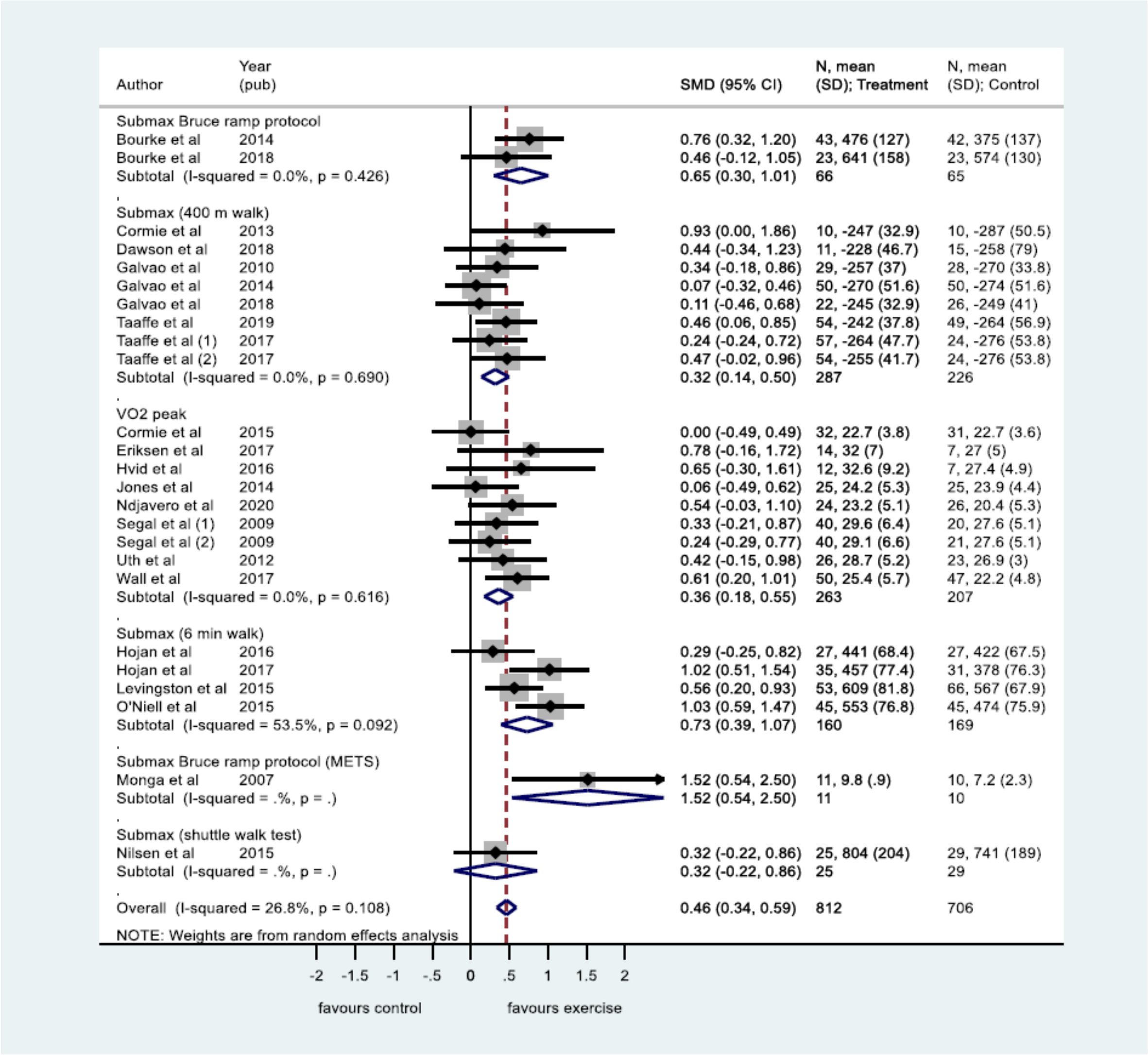


**Figure S6.** Pooled standard mean difference (SMD) on Cardiovascular fitness comparing exercise interventions with usual care or control in men with prostate cancer stratified after assessment instrument. A random effects model of DerSimonian & Laird, with estimate of heterogeneity from the Mantel-Haenszel model was used.

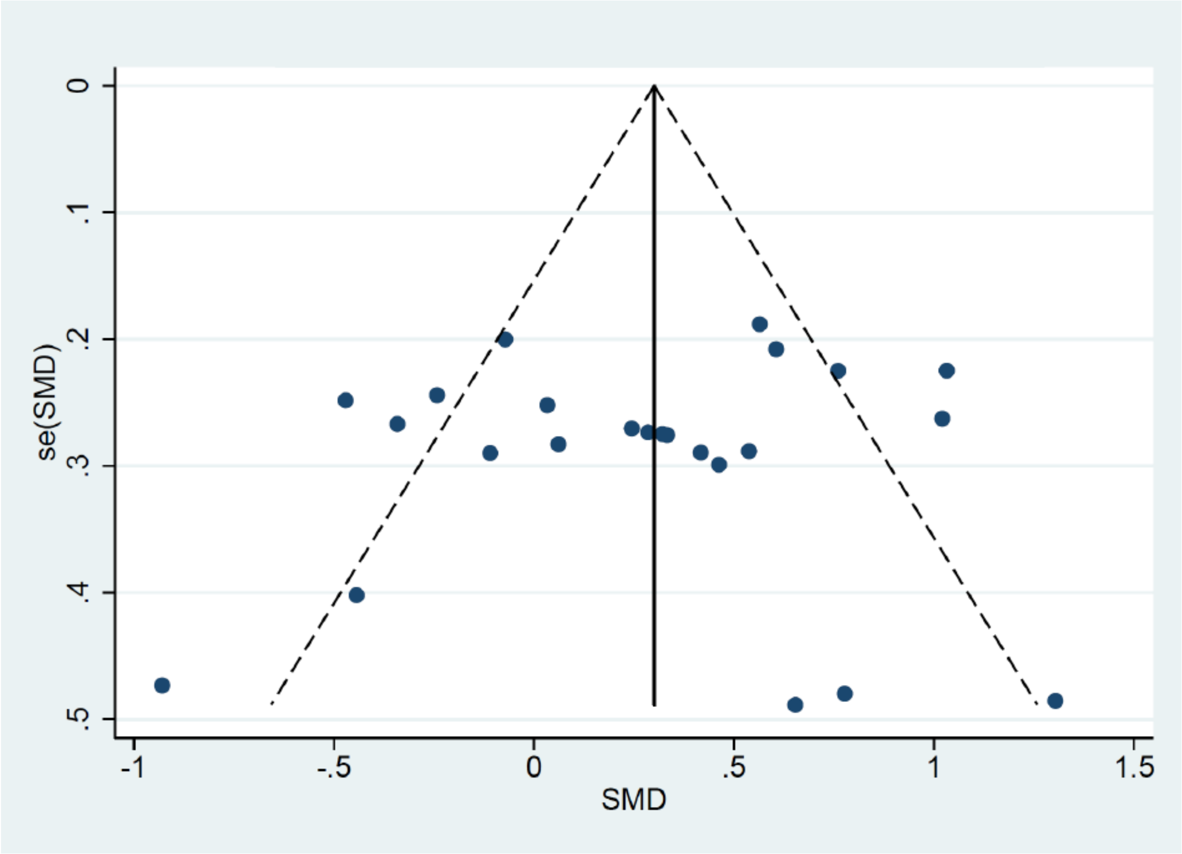


**Figure S7.** Funnel plot with pseudo 95% confidence limit on Cardiovascular function in men with prostate cancer. Egger's test showed no small-study effects (P=0.647)

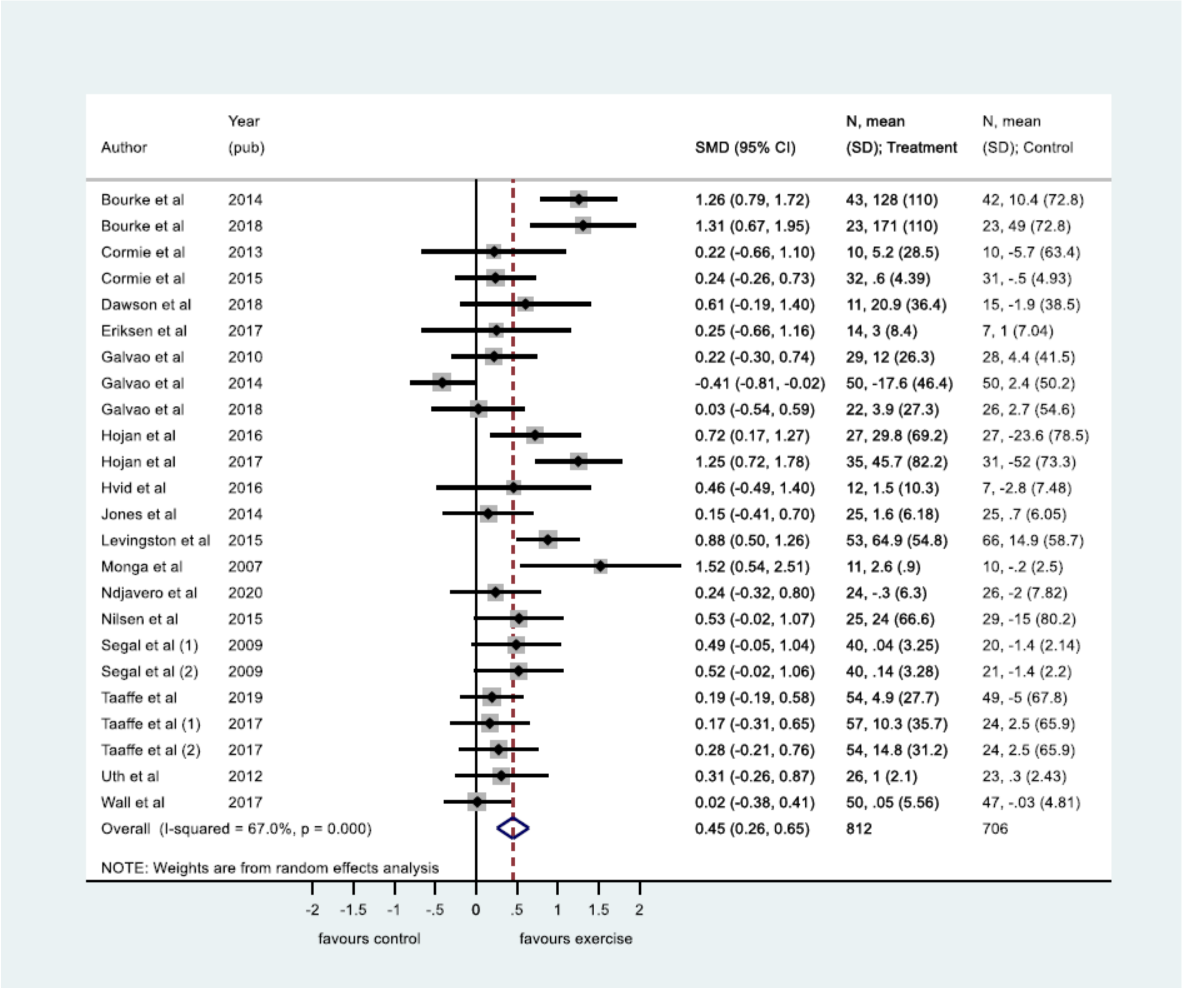


**Figure S8.** Pooled standard mean difference (SMD) for change score values on Cardiovascular fitness comparing exercise interventions with usual care or control in men with prostate cancer. A random effects model of DerSimonian & Laird, with estimate of heterogeneity from the Mantel-Haenszel model was used.
