# Supplementary material for: Do Patients with Prostate Cancer Benefit from Exercise Interventions? A Systematic Review and Meta-Analysis
Source: Int J Environ Res Public Health. 2022 Jan 15;19(2):972. doi: 10.3390/ijerph19020972 (PMC8776086; doi:10.3390/ijerph19020972)
Supplement: Supplementary file 1 [file ijerph-19-00972-s001.zip › supplementary/File S5.pdf]

Online reource 5 – remaining outcomes and additional analyses to remaining outcomes

Fatigue

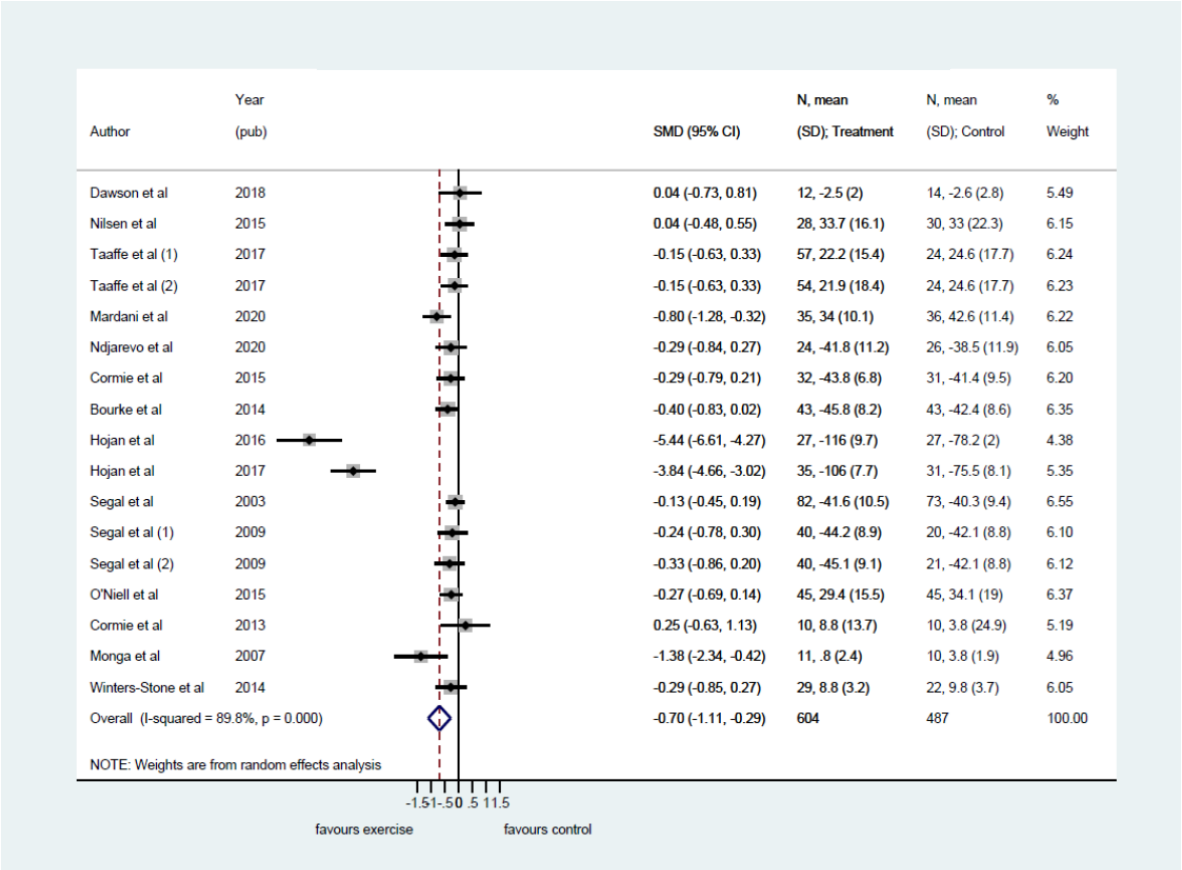

Figure S9. Pooled standard mean difference (SMD) on Fatigue comparing exercise interventions with usual care or control in men with prostate cancer. A random effects model of DerSimonian & Laird, with estimate of heterogeneity from the Mantel-Haenszel model was used.

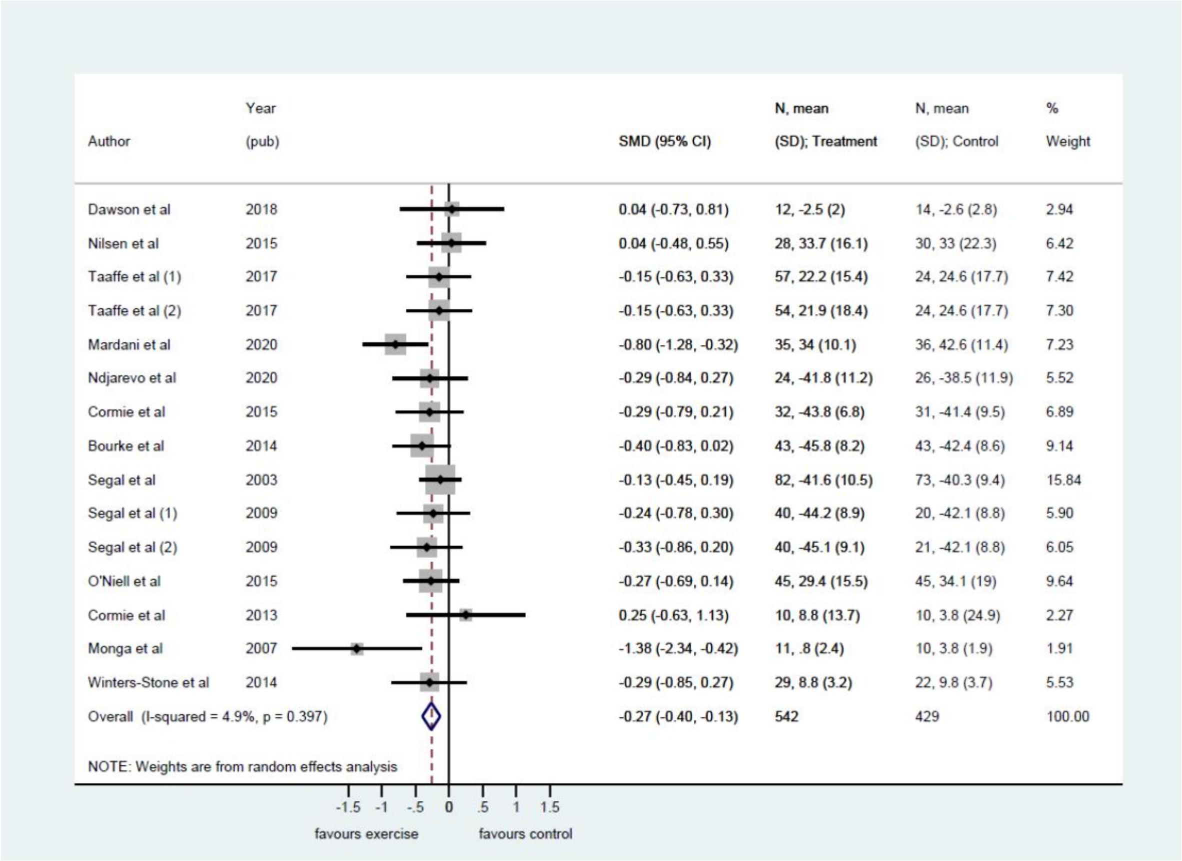

Figure S10. Pooled standard mean difference (SMD) on Fatigue comparing exercise interventions with usual care or control in men with prostate cancer. A random effects model of DerSimonian & Laird, with estimate of heterogeneity from the Mantel-Haenszel model was used. NOTE: Hojan et al. 2016 and 2017 was excluded from this meta-analysis.

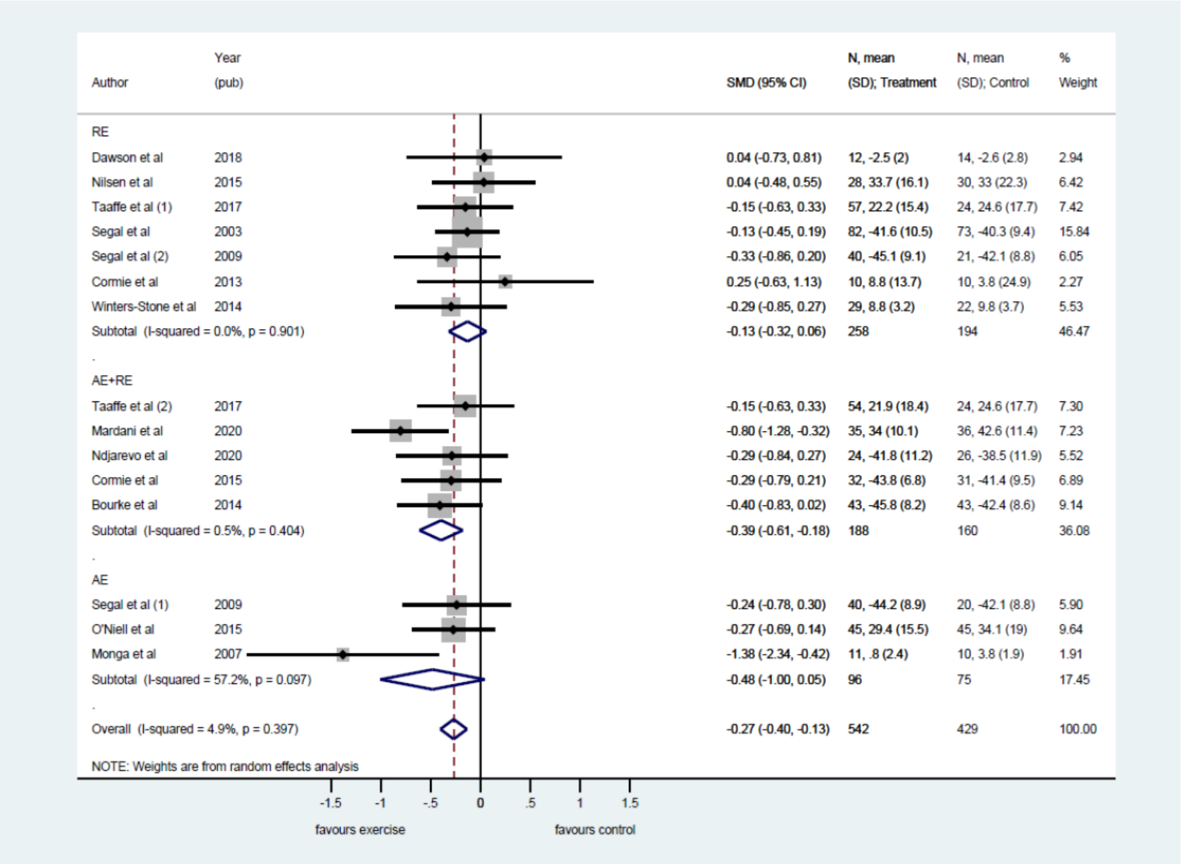

Figure S11. Pooled standard mean difference (SMD) on Fatigue comparing exercise interventions with usual care or control in men with prostate cancer stratified after exercise modality. A random effects model of DerSimonian & Laird, with estimate of heterogeneity from the Mantel-Haenszel model was used. NOTE: Hojan et al. 2016 and 2017 was excluded from this meta-analysis.

Lower body strength

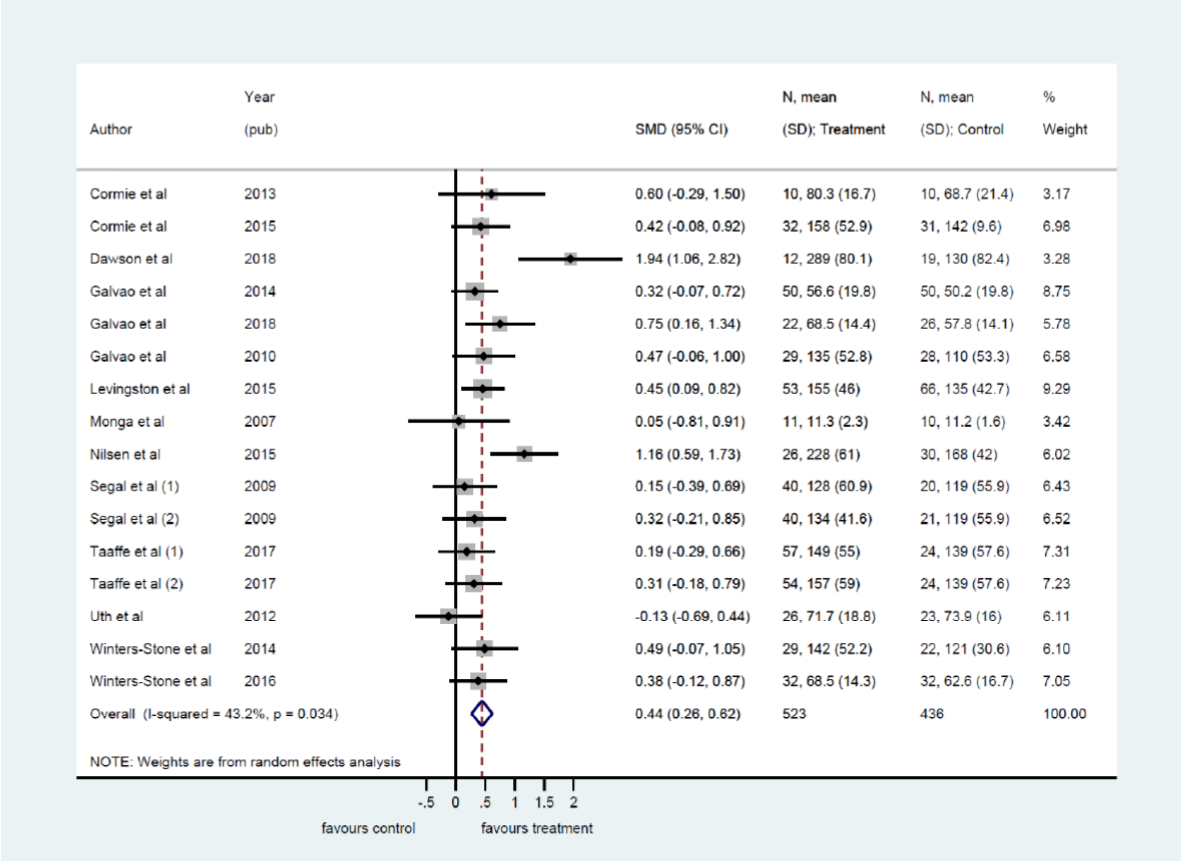

Figure S12. Pooled standard mean difference (SMD) on Lower body strength comparing exercise interventions with usual care or control in men with prostate cancer. A random effects model of DerSimonian & Laird, with estimate of heterogeneity from the Mantel-Haenszel model was used.

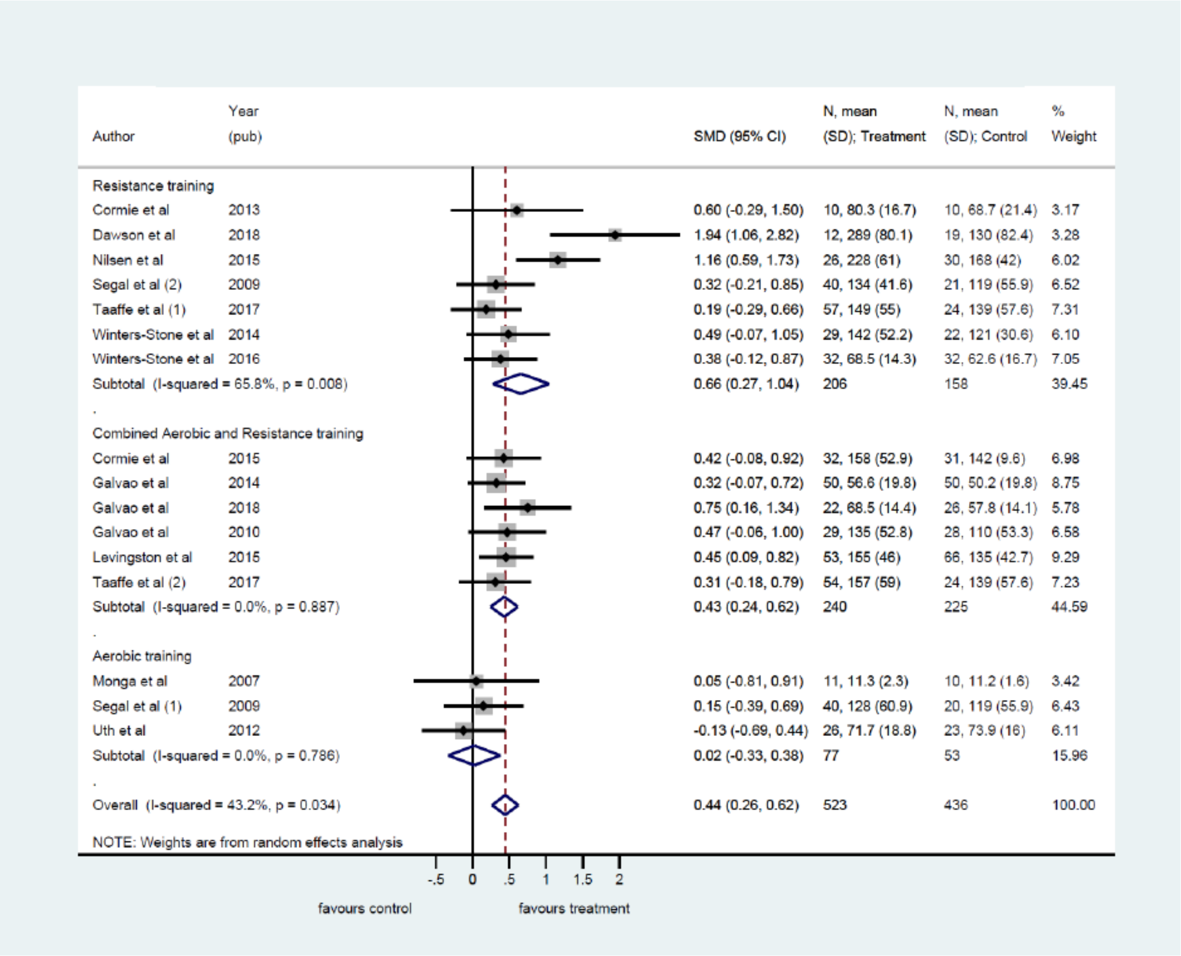

Figure S13. Pooled standard mean difference (SMD) on Lower body strength comparing exercise interventions with usual care or control in men with prostate cancer stratified after exercise modality. A random effects model of DerSimonian & Laird, with estimate of heterogeneity from the Mantel-Haenszel model was used.

Lean body mass

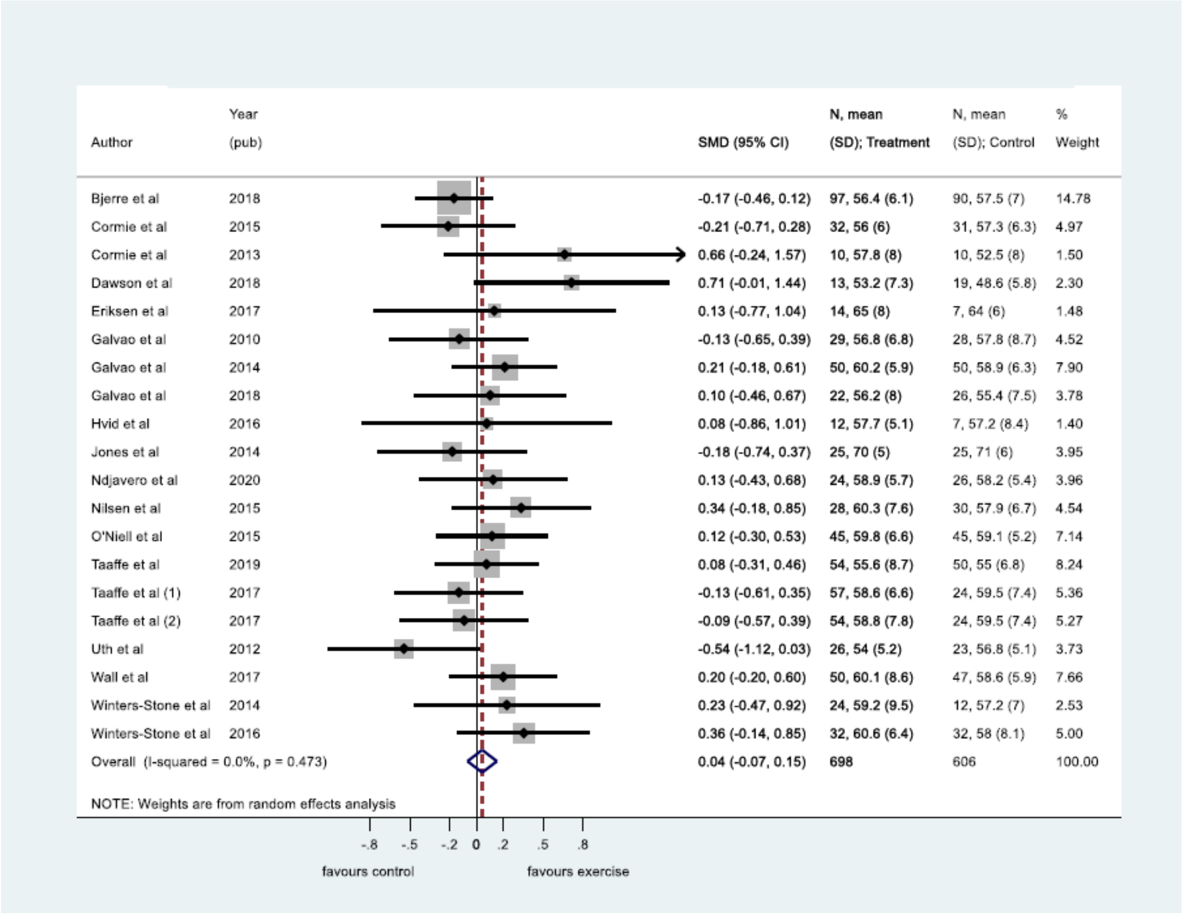

Figure S14. Pooled standard mean difference (SMD) on Lean body mass comparing exercise interventions with usual care or control in men with prostate cancer. A random effects model of DerSimonian & Laird, with estimate of heterogeneity from the Mantel-Haenszel model was used.

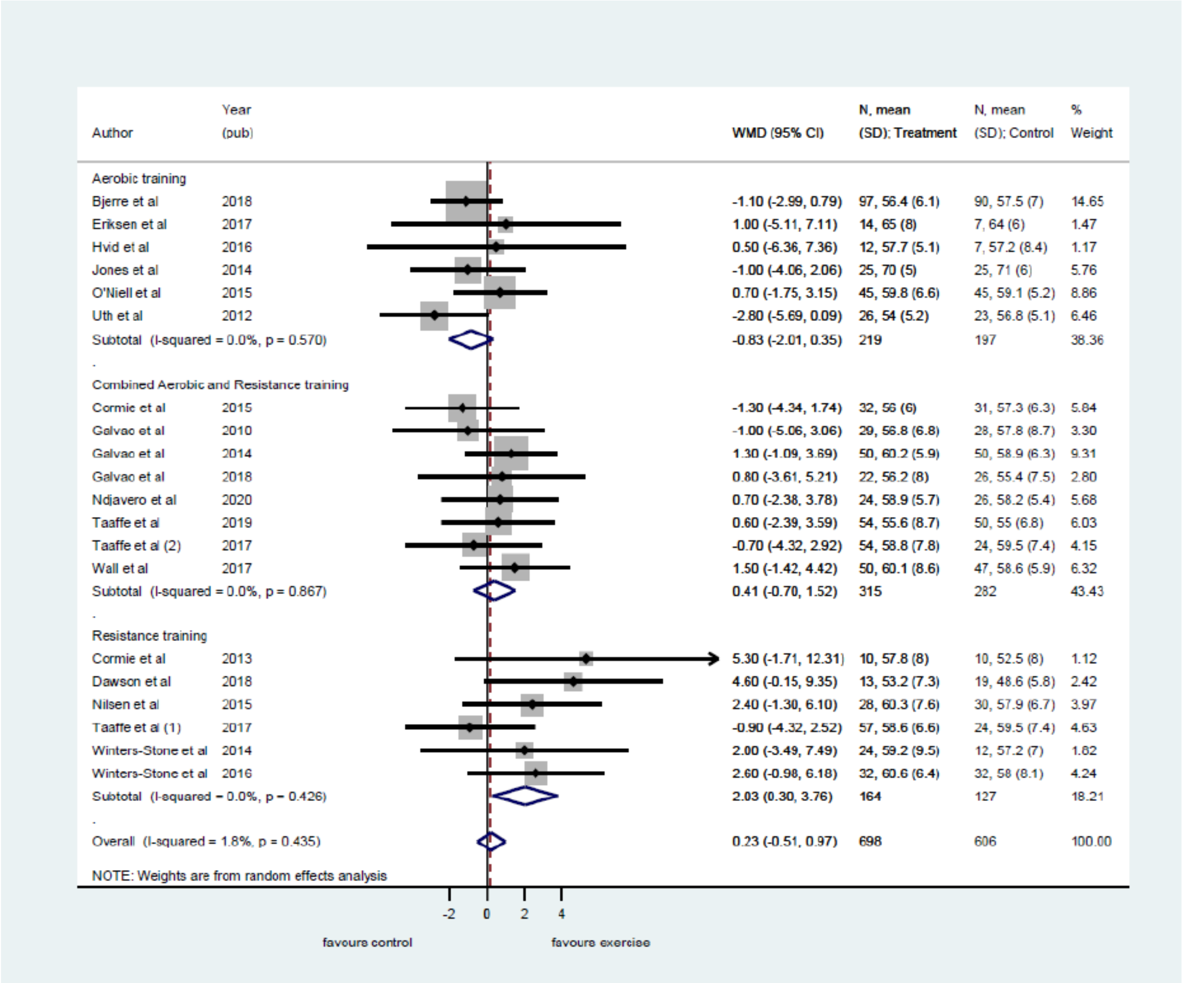

Figure S15. Pooled standard mean difference (SMD) on Lean body mass comparing exercise interventions with usual care or control in men with prostate cancer stratified after exercise modality. A random effects model of DerSimonian & Laird, with estimate of heterogeneity from the Mantel-Haenszel model was used.

Whole body fat

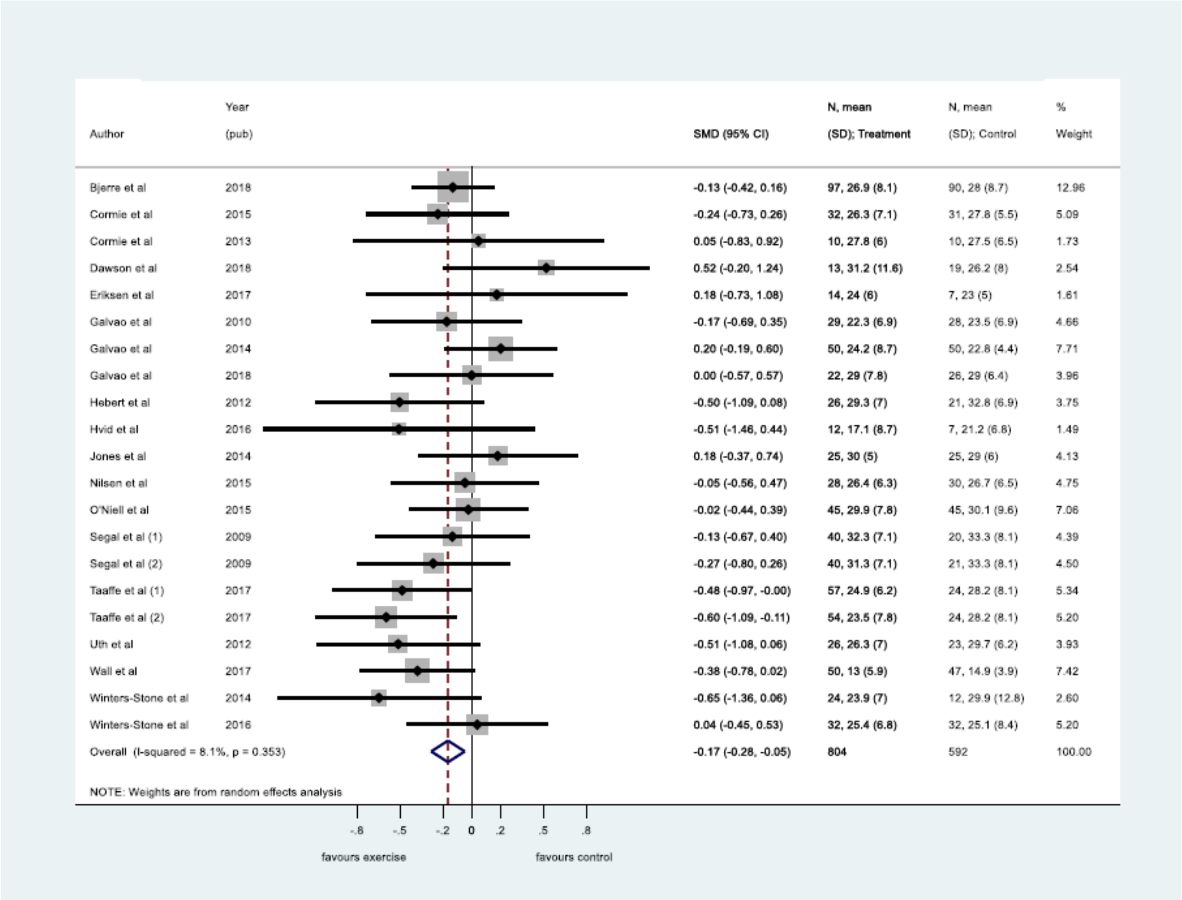

Figure S16. Pooled standard mean difference (SMD) on Whole body fat comparing exercise interventions with usual care or control in men with prostate cancer. A random effects model of DerSimonian & Laird, with estimate of heterogeneity from the Mantel-Haenszel model was used.

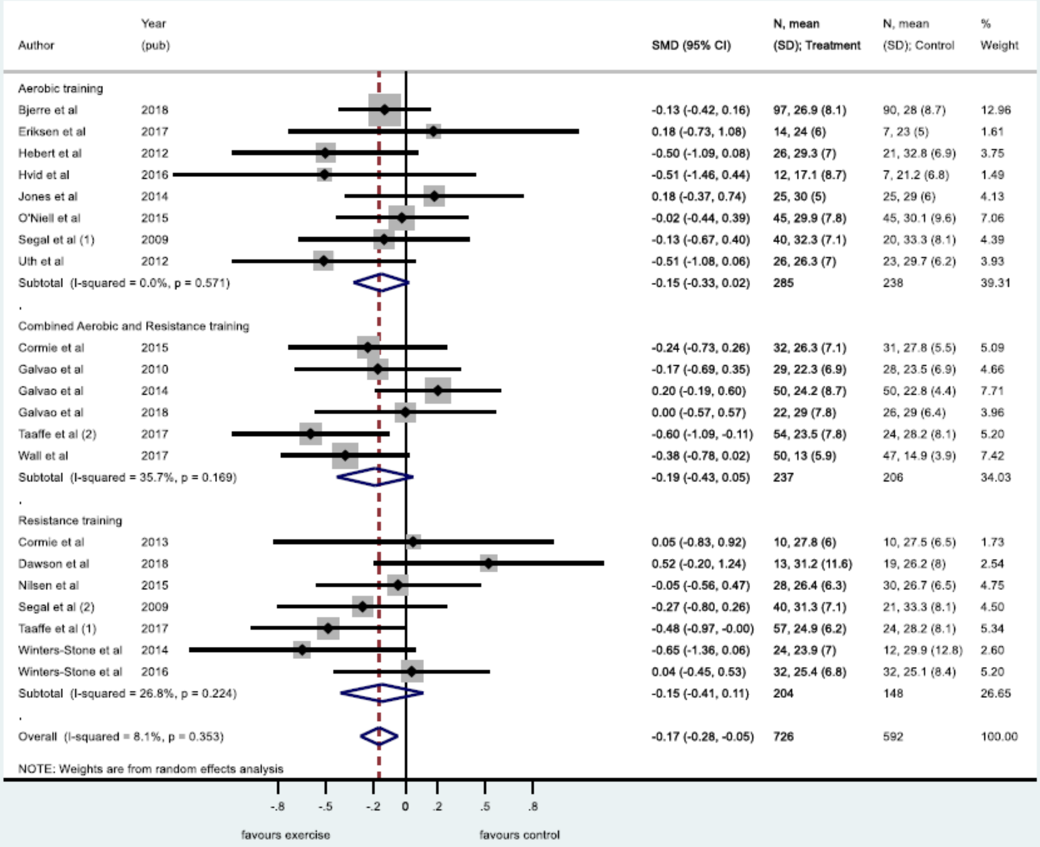

Figure S17. Pooled standard mean difference (SMD) on Whole body fat comparing exercise interventions with usual care or control in men with prostate cancer stratified after exercise modality. A random effects model of DerSimonian & Laird, with estimate of heterogeneity from the Mantel-Haenszel model was used.

General physical health

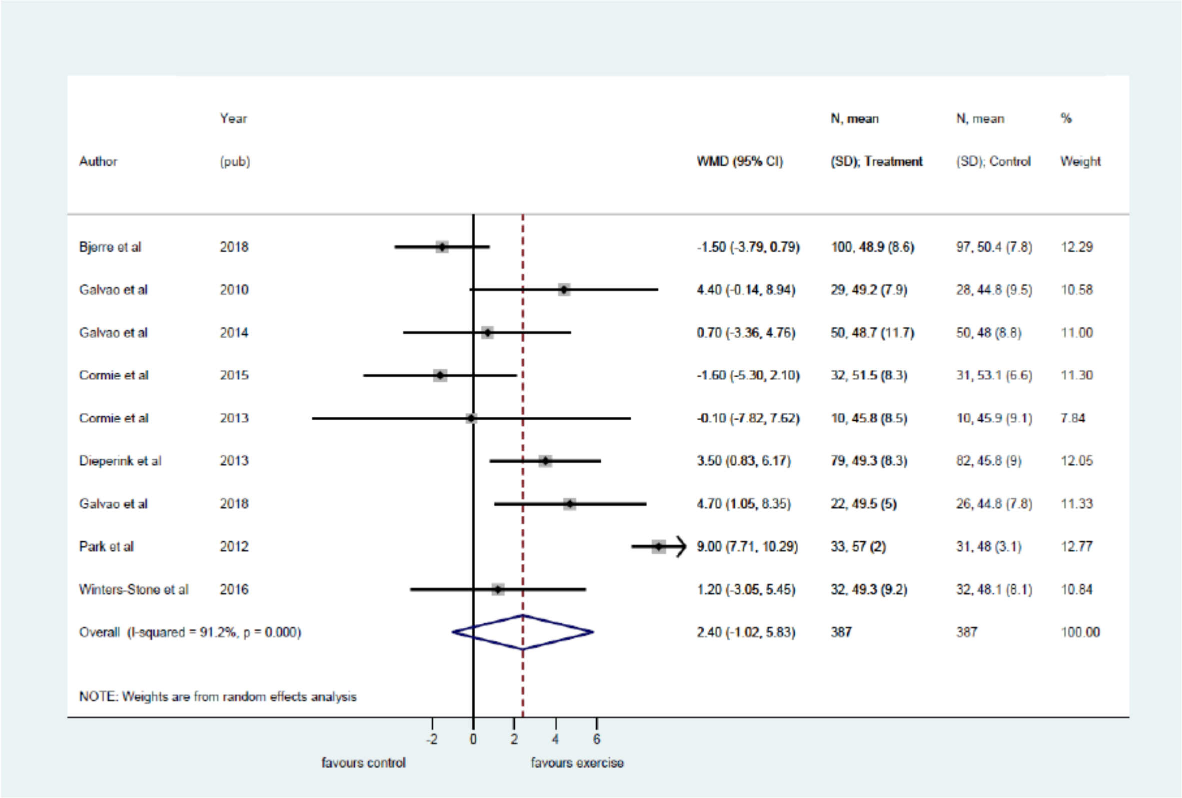

Figure S18. Pooled standard mean difference (SMD) on General physical health comparing exercise interventions with usual care or control in men with prostate cancer. A random effects model of DerSimonian & Laird, with estimate of heterogeneity from the Mantel-Haenszel model was used.

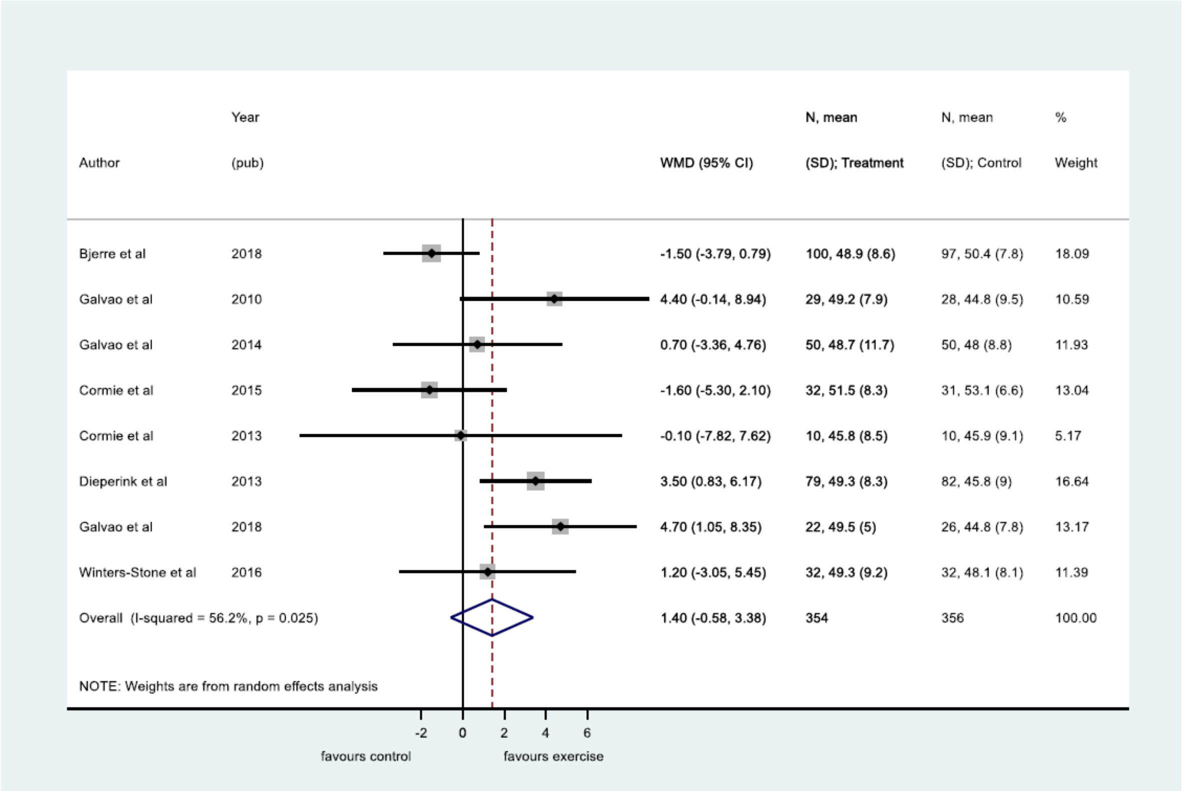

Figure S19. Pooled standard mean difference (SMD) on General physical health comparing exercise interventions with usual care or control in men with prostate cancer. A random effects model of DerSimonian &

Laird, with estimate of heterogeneity from the Mantel-Haenszel model was used. NOTE: Park et al. 2012 was excluded from the meta-analysis.

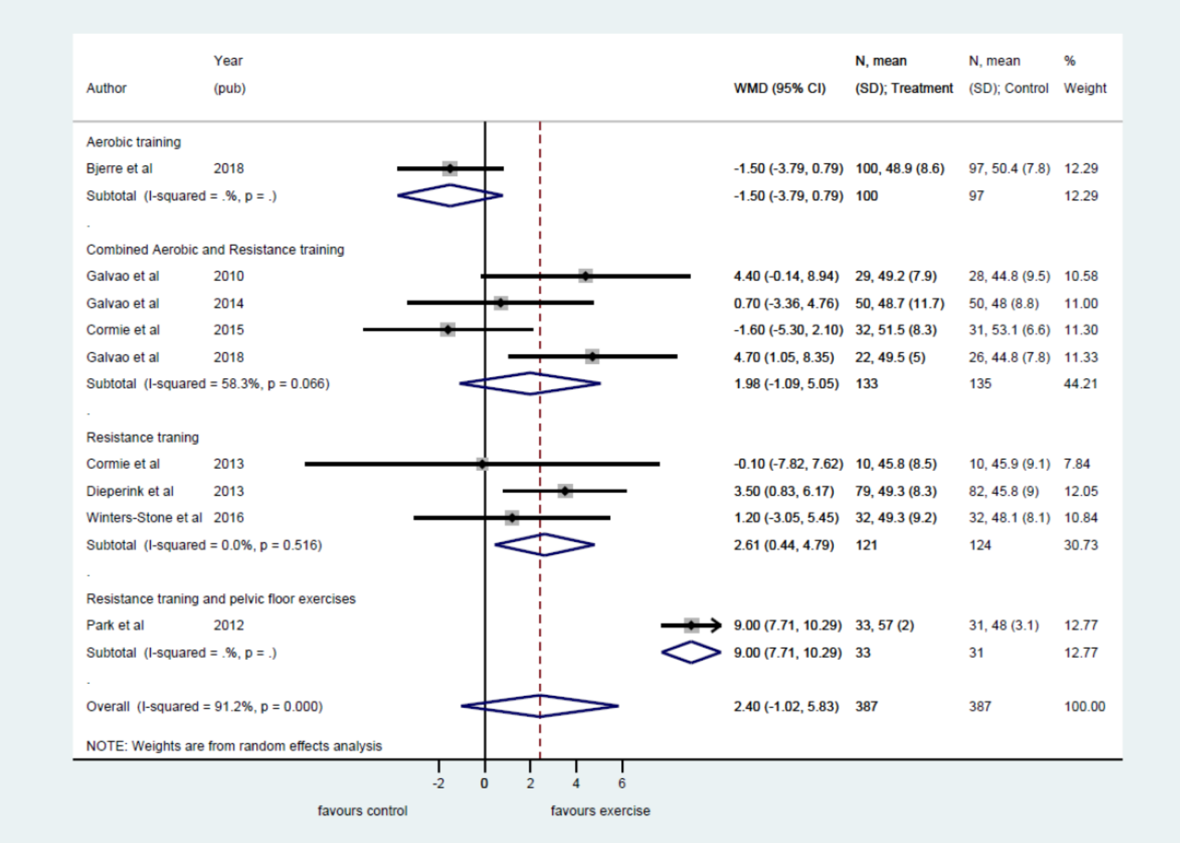

Figure S20. Pooled standard mean difference (SMD) on General physical health comparing exercise interventions with usual care or control in men with prostate cancer stratified after exercise modality. A random effects model of DerSimonian & Laird, with estimate of heterogeneity from the Mantel-Haenszel model was used.

General mental health

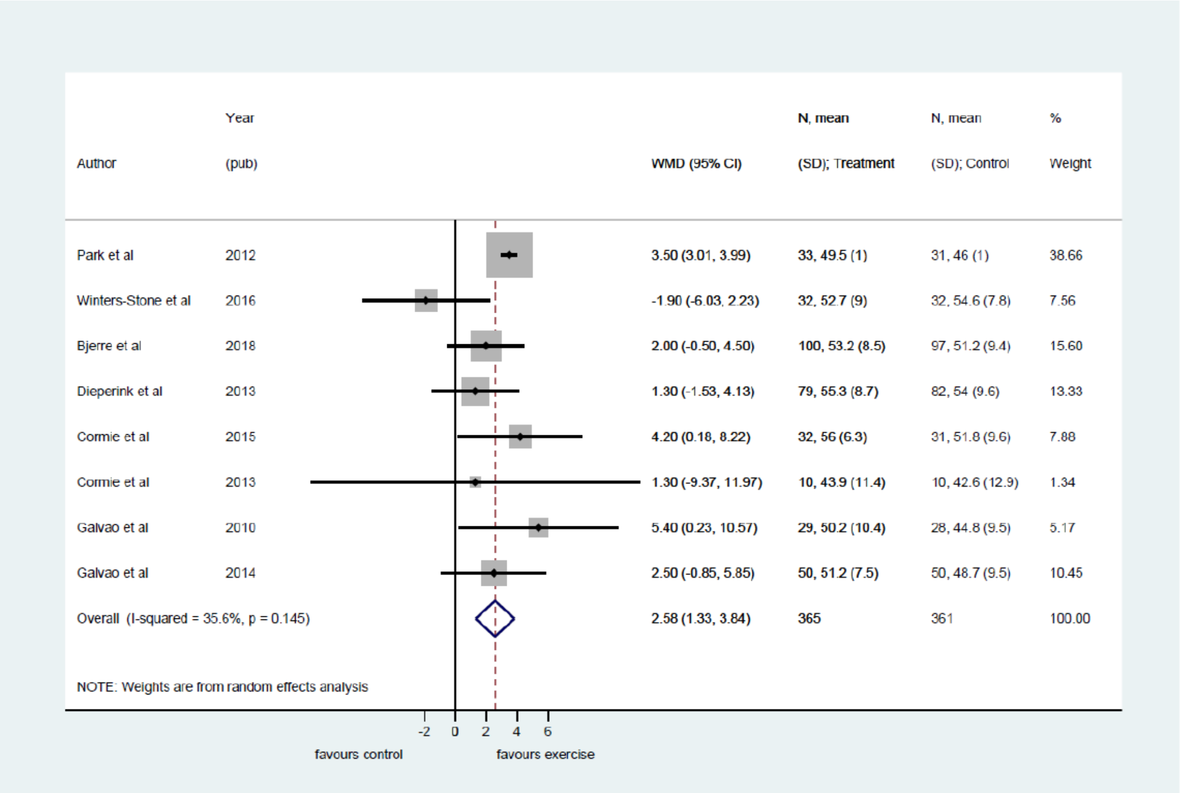

Figure S21. Pooled standard mean difference (SMD) on General mental health comparing exercise interventions with usual care or control in men with prostate cancer. A random effects model of DerSimonian & Laird, with estimate of heterogeneity from the Mantel-Haenszel model was used.

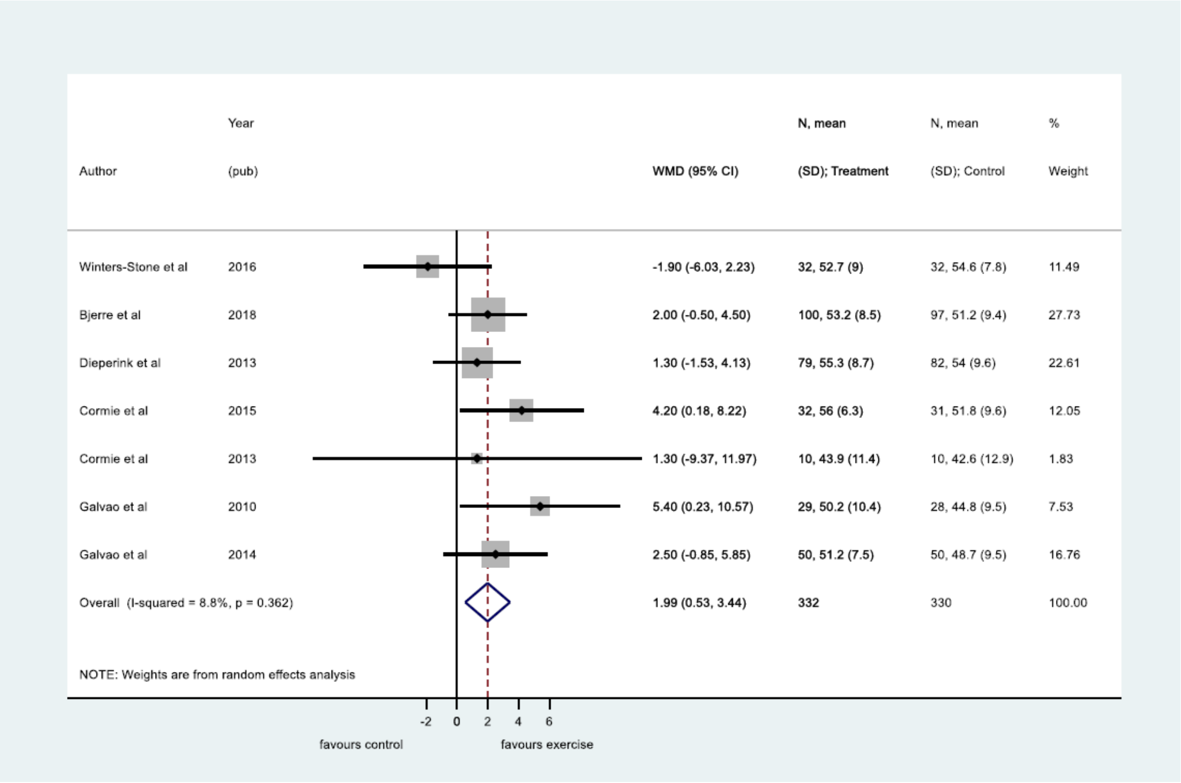

Figure S22. Pooled standard mean difference (SMD) on General mental health comparing exercise interventions with usual care or control in men with prostate cancer. A random effects model of DerSimonian & Laird, with

estimate of heterogeneity from the Mantel-Haenszel model was used. NOTE: Park et al. 2012 was excluded from the meta-analysis.

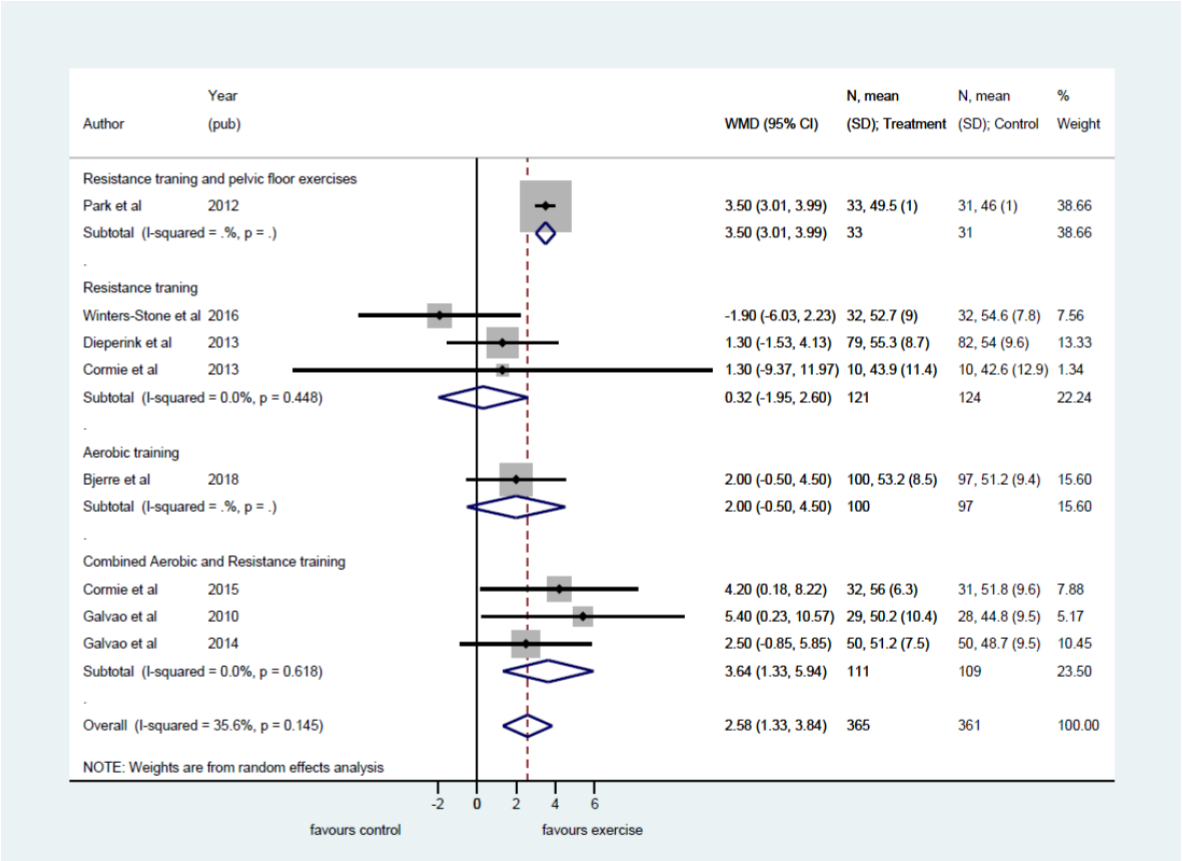

Figure S23. Pooled standard mean difference (SMD) on General mental health comparing exercise interventions with usual care or control in men with prostate cancer stratified after exercise modality. A random effects model of DerSimonian & Laird, with estimate of heterogeneity from the Mantel-Haenszel model was used.

Systolic blood pressure

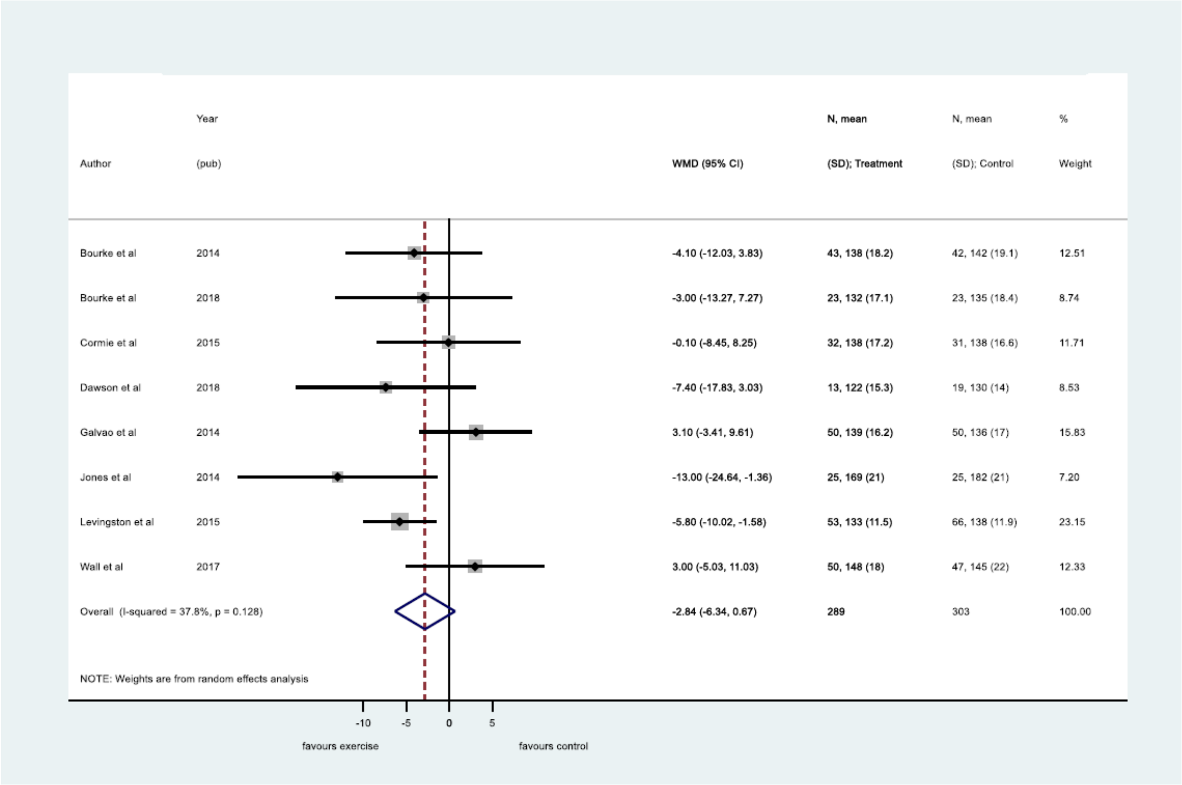

Figure S24. Pooled standard mean difference (SMD) on Systolic blood pressure comparing exercise interventions with usual care or control in men with prostate cancer. A random effects model of DerSimonian & Laird, with estimate of heterogeneity from the Mantel-Haenszel model was used.

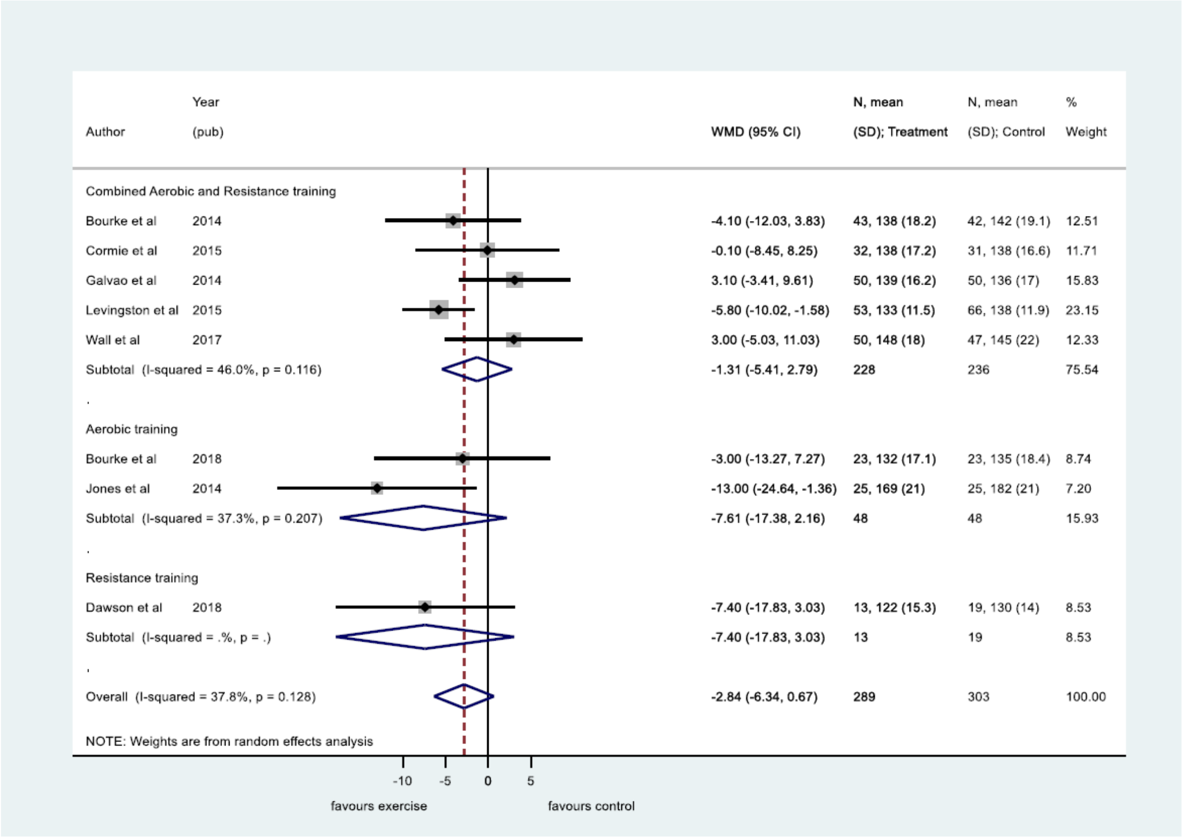

Figure 25. Pooled standard mean difference (SMD) on Systolic blood pressure comparing exercise interventions with usual care or control in men with prostate cancer stratified after exercise modality. A random effects model of DerSimonian & Laird, with estimate of heterogeneity from the Mantel-Haenszel model was used.

Diastolic blood pressure

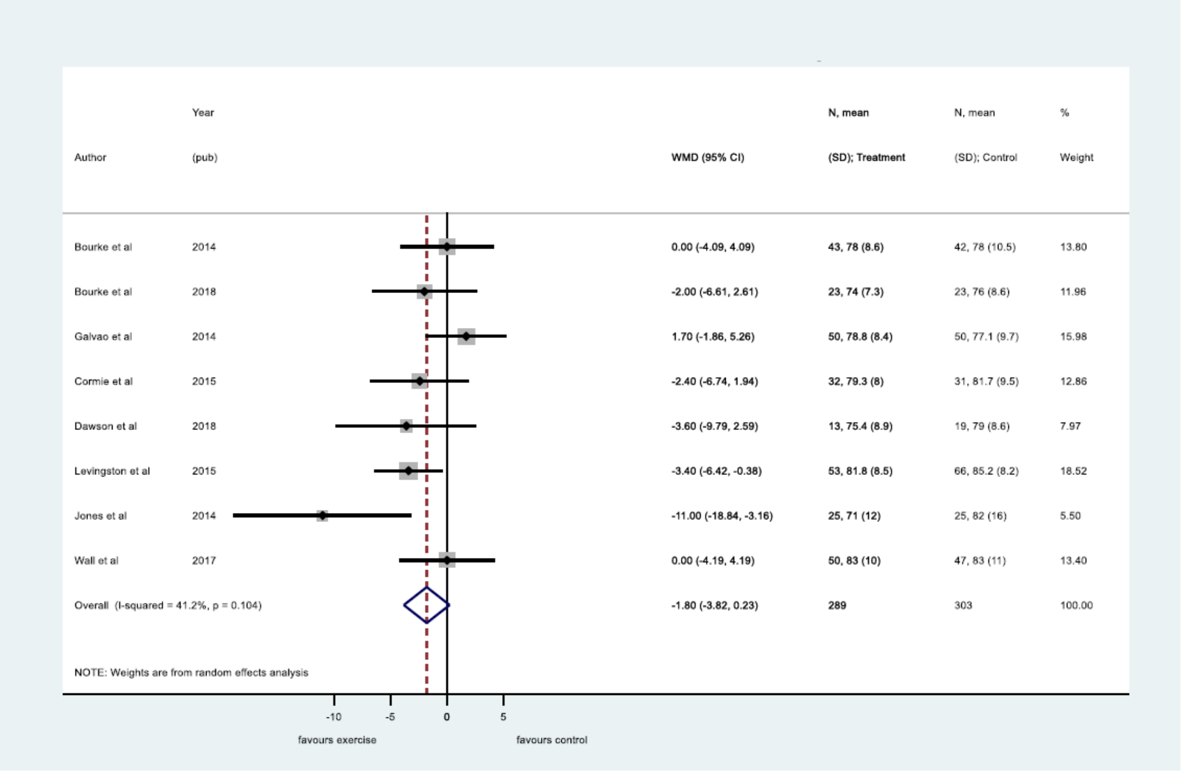

Figure S26. Pooled standard mean difference (SMD) on Diastolic blood pressure comparing exercise interventions with usual care or control in men with prostate cancer. A random effects model of DerSimonian & Laird, with estimate of heterogeneity from the Mantel-Haenszel model was used.

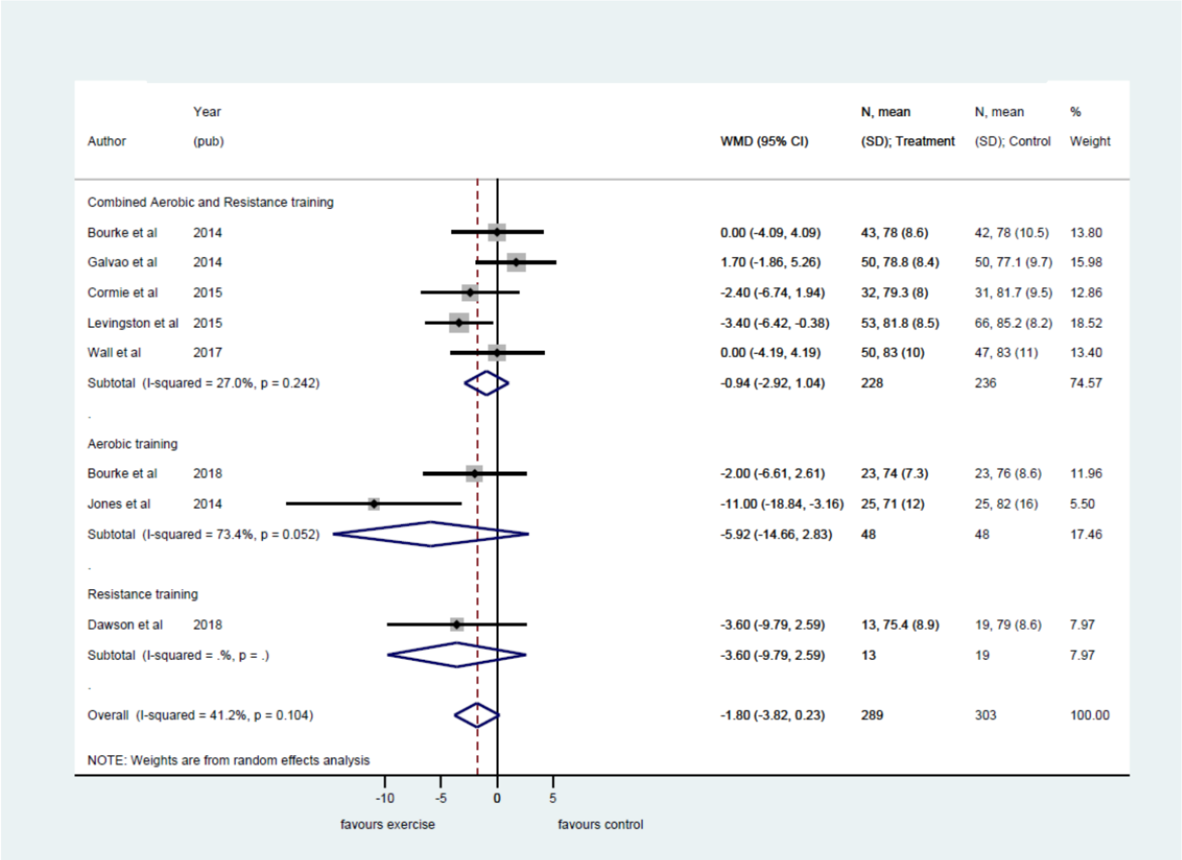

Figure S27. Pooled standard mean difference (SMD) on Diastolic blood pressure comparing exercise interventions with usual care or control in men with prostate cancer stratified after exercise modality. A random effects model of DerSimonian & Laird, with estimate of heterogeneity from the Mantel-Haenszel model was used.
